# Supplementary material for: Structural insights into sodium transport by the oxaloacetate decarboxylase sodium pump
Source: eLife. 2020 May 27;9:e53853. doi: 10.7554/eLife.53853 (PMC7274780; doi:10.7554/eLife.53853)
Supplement: Supplementary file 1. — (A) Crystal diffraction data collection and structure refinement statistics.(B) Previous mutagenesis studies performed on KpOAD. (C) Summary of ITC experiments at different pH. [file elife-53853-supp1.docx]

Supplementary file 1A. Crystal diffraction data collection and structure refinement statistics

|  | Crystal structure of the *St*OAD βγ sub-complex (PDB 6IVA) |
| --- | --- |
| **Data collection** |  |
| Space group | C222_1_ |
| Cell dimensions |  |
| *a*, *b*, *c* (Å) | 107.47, 198.08, 241.75 |
| α, β, γ (°) | 90, 90, 90 |
| Resolution (Å) | 50.0-4.40 (4.48-4.40) * |
| *R*_merge_ | 0.160 (2.447) |
| *I*/σ*I* | 14.99 (1.53) |
| Completeness (%) | 99.8 (99.9) |
| Redundancy | 6.9 (6.4) |
|  |  |
| **Refinement** |  |
| Resolution (Å) | 50.0-4.40 (4.68-4.40) |
| No. reflections | 16,615 (2710) |
| *R*_work/_ *R*_free_ | 30.96/33.56 (40.49/39.09) |
| No. atoms |  |
| Protein | 10122 |
| Ligand/ion | 0 |
| Water | 0 |
| B-factors |  |
| Protein | 242.51 |
| R.m.s deviations |  |
| Bond lengths (Å) | 0.003 |
| Bond angles (º) | 0.717 |

*Highest resolution shell is shown in parenthesis.

Supplementary file 1B. Previous mutagenesis studies performed on *Kp*OAD

| Resi | Equ. *St*OAD resi | OAD activity (%)^1^ | C_1/2_ (mM)^2^ | Sodium pump activity | Growth rescue^3^ | Carboxyl- biotin (%)^4^ | Position in the structure |
| --- | --- | --- | --- | --- | --- | --- | --- |
| Wild type | - | 100% | 0.5 | Full | Yes | 3%/7% (2 reports) | - |
| N55 | N55 | 40% (N55A) | 1.2 (N55A) |  | Yes (N55A) |  | In T2, in the periplasmic face, side chain hydrogen bonds with the Leu329 main chain carbonyl in T8 |
| T148 | T148 | 10% (T148A) | 0.7 (T148A) |  | Yes (T148A) |  | In the T3-αH1 loop, in the cytoplasmic face, side chain points into the protein interior |
| D149 | D149 | ~100% (D149E/Q) |  | Retained (D149Q) |  | 4% (D149Q) | In the T3-αH1 loop, in the cytoplasmic face, side chain solvent exposed |
| T189 | T189 | 30% (T189A) | 1.2 (T189A) |  | Yes (T189A) |  | In the N-terminus of helical hairpin 1, in the periplasmic face, side chain solvent exposed |
| Q192 | Q192 | 5% (Q192L) | 3.6 (Q192L) |  | Yes (Q192L) |  | In helical hairpin 1, in the periplasmic face, side chain solvent exposed |
| G197 | G197 | 20% (G197A) | 0.8 (G197A) |  | Yes (G197A) |  | In helical hairpin 1, in the protein interior in a hydrophobic environment |
| G200 | G200 | 10% (G200A) | 0.7 (G200A) |  | Yes (G200A) |  | In helical hairpin 1, in the protein interior, close to the tip |
| G201 | G201 | 20% (G201A) | 2.3 (G201A) |  | Yes (G201A) |  | In helical hairpin 1 at the tip |
| D203 | D203 | 0% (D203N/E) |  | Lost (D203N/E) |  | 98% (D203N) | In helix hairpin 1 at the tip, likely contribute to sodium binding |
| G204 | G204 | 15% (G204A) | 1.9 (G204A) |  | Yes (G204A) |  | In helix hairpin 1 at the tip, interacts with Y227 |
| T206 | T206 | 40% (T206A) | 0.8 (T206A) |  | Yes (T206A) |  | In helix hairpin 1, in the protein interior in a hydrophobic environment |
| Y209 | Y209 | 8% (Y209A) | 1.7 (Y209A) |  | Yes (Y209A) |  | In helix hairpin 1, side chain exposed to periplasm |
| E217 | E217 | 12% (E217A) | 0.9 (E217A) |  | Yes (E217A) |  | In the loop between helical hairpin 1 and T5, side chain exposed to periplasm |
| Y227 | Y227 | 1% (Y227A), 30% (Y227C), 20% (Y227F) | 1.1 (Y227C), 1.0 (Y227F) |  | No (Y227A), Yes (Y227C/F) | 9% (Y227A) | In T5, side chain mostly buried in the protein interior in a hydrophobic environment |
| Y229 | Y229 | 0.05% (Y229A), 0% (Y229F) |  |  | No (Y229A/F) | 9% (Y229A), ~100% (Y229F) | In T5, side chain exposed to the membrane bilayer, forms a hydrogen bond with the L343 main chain carbonyl in T9 |
| Q237 | Q237 | 15% (Q237A) | 0.7 (Q237A) |  | Yes (Q237A) |  | In T5 in the protein interior, side chain hydrogen bonds to the Ser378 side chain in helical hairpin 2 |
| D282 | D282 | 50% (D282A) | 0.8 (D282A) |  | Yes (D282A) |  | In the T6-T7 loop, side chain exposed to periplasm |
| C291 | C291 | 10% (C291E) |  |  | Yes (C291E) |  | In T7 in the protein interior in a hydrophobic environment |
| T318 | T318 | 30% (T318A) | 2.7 (T381A) |  | Yes (T318A) |  | In T8, side chain exposed to cytoplasm |
| C351 | G351 | 8% (C351E) |  |  | Yes (C351E) |  | In T9, exposed to lipid bilayer |
| T354 | T354 | 8% (T354A) | 0.9 (T354A) |  | Yes (T354A) |  | In T9, side chain points to the protein interior in a hydrophobic environment |
| N373 | N373 | 0.7% (N373L) | 0.7 (N373L) |  | No (N373L) |  | N-terminal to helical hairpin 2, side chain points to the protein interior, hydrogen bonds to the E249 mainchain C-terminal to T5 |
| G377 | G377 | 0% (G377A) |  |  | No (G377A) | 100% (G377A) | In helical hairpin 2, cannot accommodate a side chain here |
| G380 | G380 | 39% (G380A) | 0.1 (G380A) |  | Yes (G380A) |  | In helical hairpin 2, packs against G163, in the protein interior in a hydrophobic environment |
| S382 | S382 | 0% (S382A/C/E/N/Q), ~10% (S382T/D) | 0.8 (S382D), 0.7 (S382T) |  | No (S382A/C/E/N/Q), yes (S382T/D) | 100% (S382A/E/N/Q), 60% (S382C) | On the tip of helical hairpin 2, likely contribute to sodium binding |
| R389 | R389 | 10% (R389A/L), <1% (R389D), 70% (R389K) | 1.0 (R389A), 1.3 (R389K), 2.6 (R389L) |  | No (R389A/D/L), yes (R389K) |  | In helical hairpin 2, side chain exposed to cytoplasm |
| N392 | N392 | 70% (N392L) | 0.6 (N392L) |  | Yes (N392L) |  | In helical hairpin 2, side chain exposed to cytoplasm |
| S419 | S419 | 10% (S419A) | 1.1 (S419A) |  | Yes (S419A) |  | In T10 near the tip of helical hairpin 1, may contribute to sodium binding |

^1^ Activity of the wild type OAD is 100%.

^2^ Sodium concentration for half-maximum OAD activity.

^3^ Growth of the *E. coli* EP423 strain in high salt environment.

^4^ Carbon-14-labelling for carboxyl-biotin.

Supplementary file 1C. Summary of ITC experiments at different pH^1^

| WT | | | | | | |
| --- | --- | --- | --- | --- | --- | --- |
| Buffer | pH | *K*_d_ (mM) | K (M^-1^) | ΔH (cal/mol) | ΔS (cal/mol/deg) | -ΔH _(H+)_ (cal/mol)^2^ |
| Mes | 5.55 | 1.99 ± 0.02 | 502 ± 6 | -3679 ± 22 | 0.0178 | 3537 |
| Mes | 5.75 | 1.71 ± 0.03 | 585 ± 11 | -3885 ± 37 | -0.36 | 3537 |
| Tris | 8.45 | 0.69 ± 0.02 | 1440 ± 40 | -3240 ± 37 | 3.59 | 11341 |
| Citrate | 5.79 | 1.61 ± 0.04 | 623 ± 16 | -3205 ± 43 | 2.03 | -807 |
| Mes | 5.79 | 1.59 ± 0.03 | 630 ± 13 | -3375 ± 35 | 1.50 | 3537 |
| Bis-Tris | 5.79 | 1.51 ± 0.02 | 664 ± 7 | -3479 ± 19 | 1.24 | 6788 |
| Pipes | 7.2 | 1.36 ± 0.06 | 737 ± 31 | -2320 ± 48 | 5.34 | 2677 |
| Hepes | 7.2 | 0.97 ± 0.03 | 1030 ± 34 | -3426 ± 52 | 2.29 | 4876 |
| Tris | 7.2 | 0.96 ± 0.03 | 1040 ± 31 | -3371 ± 46 | 2.49 | 11341 |
| Hepes | 8.2 | 0.83 ± 0.02 | 1200 ± 32 | -2725 ± 32 | 4.96 | 4876 |
| Tricine | 8.2 | 0.82 ± 0.01 | 1220 ± 18 | -3395 ± 21 | 2.75 | 7498 |
| Tris | 8.2 | 0.75 ± 0.02 | 1340 ± 36 | -2709 ± 30 | 5.22 | 11341 |
| E40A | | | | | | |
| Buffer | pH | *K*_d_ (mM) | K (M^-1^) | ΔH (cal/mol) | ΔS (cal/mol/deg) | -ΔH _(H+)_ (cal/mol) |
| Mes | 5.55 | 3.16 ± 0.12 | 316 ± 12 | -2741 ± 60 | 2.36 | 3537 |
| Mes | 5.75 | 2.75 ± 0.05 | 364 ± 7 | 2351 ± 25 | 3.84 | 3537 |
| Tris | 8.45 | 1.52 ± 0.05 | 659 ± 23 | -1543 ± 28 | 7.72 | 11341 |
| Citrate | 5.79 | 2.26 ± 0.07 | 443 ± 13 | -2906 ± 47 | 2.36 | -807 |
| Mes | 5.79 | 2.25 ± 0.04 | 444 ± 9 | -2983 ± 32 | 2.11 | 3537 |
| Bis-Tris | 5.79 | 2.24 ± 0.02 | 446 ± 5 | -2581 ± 15 | 3.46 | 6788 |
| Pipes | 7.2 | 2.34 ± 0.07 | 427 ± 13 | -2453 ± 40 | 3.81 | 2677 |
| Hepes | 7.2 | 2.19 ± 0.08 | 457 ± 18 | -2197 ± 46 | 4.80 | 4876 |
| Tris | 7.2 | 2.16 ± 0.09 | 464 ± 20 | -1944 ± 45 | 5.68 | 11341 |
| Hepes | 8.2 | 1.93 ± 0.07 | 519 ± 18 | -1590 ± 29 | 7.09 | 4876 |
| Tricine | 8.2 | 1.63 ± 0.04 | 612 ± 15 | -3110 ± 39 | 2.32 | 7498 |
| Tris | 8.2 | 1.59 ± 0.05 | 628 ± 21 | -1337 ± 22 | 8.32 | 11341 |

^1^Errors were derived from fitting the data to the one set of sites model.

^2^ΔH_(H_+_)_ refers to the intrinsic protonation enthalpy of the buffer.
